# Complementary and Alternative Medicine and Osteoarthritis

**DOI:** 10.5772/56431

**Published:** 2013-03-22

**Authors:** Chenchen Wang

**Affiliations:** Associate Professor of Medicine, Director of the Center for Complementary and Integrative Medicine, Tufts Medical Center/Tufts University School of Medicine, Boston, MA, USA

**Keywords:** Osteoarthritis, Mind-Body Medicine, Complementary And Alternative Medicine, Tai Chi, Acupuncture, Pain Management

## Abstract

Patients with osteoarthritis experience high levels of pain, psychological distress and have limited therapeutic options. Emerging evidence from clinical trials suggests that both acupuncture and Tai Chi mind-body therapies are safe and effective treatments for osteoarthritis.

Acupuncture has effects over and above those of ‘sham acupuncture’ and the most robust evidence to date demonstrates that acupuncture does have short-term benefits and is a reasonable referral option for patients with symptomatic osteoarthritis. Tai Chi is a mind-body exercise that enhances cardiovascular fitness, muscular strength, balance, and physical function. It also appears to be associated with reduced stress and anxiety and depression, as well as improved quality of life. Thus, Tai Chi may be safely recommended to patients with osteoarthritis as a complementary and alternative medical approach to affect patient well-being.

Integrative approaches combine the best of conventional medicine and complementary and alternative medicine to ultimately improve patient care. These modalities may lead to the development of better disease modifying strategies that could improve symptoms and decrease the progression of osteoarthritis. This overview synthesizes the current body of knowledge about Chinese mind-body medicine to better inform clinical decision-making for our rheumatic patients.

## 1. Introduction

Symptomatic osteoarthritis (OA) is the most frequent cause of dependency in lower limb tasks among ageing populations and is associated with substantial physical and psychosocial disability, reduced quality of life and substantial health care costs [1].

Currently, no effective disease-modifying remedies are available for OA. Complementary and alternative medicine (CAM) therapies have been heavily advertised and an increasing number of chronic pain patients report utilizing these modalities [2].

Clinical trials and observational studies provide encouraging evidence that acupuncture, as well as other types of mind-body therapies, offers benefits for patients with arthritis. This article reviews the current body of knowledge on the therapeutic benefits of CAM for pain and symptom relief in patients with OA to better inform clinical decision-making.

## 2. Acupuncture for Osteoarthritis

Acupuncture, originating in China more than 3,000 years ago, is one of the most popular sensory stimulation therapies. The practice is an ancient technique of inserting and manipulating fine needles to stimulate specific anatomic points, also known as acupuncture points or meridian points, on the body to facilitate the recovery of health [3]. Each year, an estimated 3 million adults in the United States receive acupuncture treatment for chronic pain, most frequently associated with OA [4].

Numerous randomized, controlled trials and more than 11 systematic reviews and meta-analyses have examined the clinical efficacy of acupuncture in patients with OA. Evidence from these trials indicates that acupuncture has some efficacy in the relief of pain. For example, an early, large, high-quality trial concluded that acupuncture significantly improved pain and function when compared with sham acupuncture (needling at specified non-acupuncture points) or health education among patients with knee OA [5].

A multicenter, randomized, controlled trial in Germany found acupuncture plus routine care were associated with marked clinical improvement in patients with chronic OA-associated pain of both the knee and hip [6]. Subsequently, another large German trial showed that both traditional Chinese acupuncture and sham acupuncture improved pain and functionality in patients with knee OA more than standard therapy. However, no difference was observed between traditional Chinese acupuncture and sham therapy [7]. Similarly, a recent trial found that traditional acupuncture was not superior to sham acupuncture; however, the providers’ practice style affected both pain reduction and satisfaction with the treatment. This finding suggests that the analgesic benefits of acupuncture may be partially mediated by the providers’ behaviour and may be enhanced by outcome expectations [8].

Three recent reviews including a systematic review, a Cochrane review and a meta-analysis evaluated the effects of acupuncture in osteoarthritis compared to no acupuncture and sham acupuncture with mixed results. A systematic review concluded that acupuncture is significantly superior to sham acupuncture in improving pain and function in patients with OA [9]. The latest Cochrane review with 16 randomized trials indicated that when compared with waiting-list controls, acupuncture recipients showed statistically and clinically significant short-term improvements in pain and function for OA. In comparison with sham controls, acupuncture provided small, statistically significant improvements [10]. In contrast, a meta-analysis observed that sham-controlled trials demonstrated clinically irrelevant short-term benefits of acupuncture, while wait list controlled trials had clinically relevant short-term benefits in pain and function, suggesting that placebo or expectation effects may be involved [11].

To address the considerable controversy, a meta-analysis of individual patient data from 29 randomized, controlled trials of acupuncture for chronic pain was recently conducted [12]. Of 19 OA studies, 6 were sham controlled, 8 had no acupuncture control, and 5 were 3-armed studies, including both sham and non-acupuncture control. Patients had access to analgesics and other standard treatments for pain. The authors report that patients receiving acupuncture had less pain than controls and traditional acupuncture was statistically superior to both sham and no-acupuncture control in the treatment of OA. The study concluded that acupuncture is effective in the treatment of chronic pain, including pain associated with OA, and is therefore a reasonable referral option. Significant differences between true and sham acupuncture indicate that acupuncture is more than a placebo. Further work is needed to understand the underlying mechanisms by which acupuncture can improve clinical symptoms.

Overall, these findings are both clinically and scientifically important. Indeed, the most robust evidence to date demonstrates that acupuncture does have short-term benefits over and above those of sham acupuncture in patients with symptomatic OA. It is therefore of major importance for clinical practice and is a reasonable referral option for patients with symptomatic OA.

## 3. Tai Chi Mind-Body Therapy for Osteoarthritis

Tai Chi is a traditional Chinese mind-body exercise that has recently grown in popularity in the United States. Over the past two decades, the potential therapeutic benefits of Tai Chi for a variety of chronic conditions have been consistently reported in the literature [13]. As a complementary mind-body approach, Tai Chi may be an especially applicable treatment for older adults with OA who are often limited in the range of activities they are able to perform. The physical component provides low-impact exercise consistent with current recommendations for OA (muscle strength, balance, flexibility and aerobic cardiovascular exercise) and the mental component could address the chronic pain state through its positive effects on psychological well-being, life satisfaction and perceptions of health. Combined, the component effects may reduce pain, improve function and slow the progression of disease and disability associated with OA.

Several randomized, controlled studies have examined the effects of Tai Chi for patients with both knee and hip OA. Hartman and colleagues were among the first to conduct a prospective, randomized, controlled clinical trial to test the efficacy of 12 weeks of Tai Chi for patients with OA [14]. A total of 35 community-dwelling participants were randomly assigned either two one-hour Tai Chi sessions per week for 12 weeks or they continued with their usual care. The results of Tai Chi training significantly improved arthritic symptoms, self-efficacy, level of tension and satisfaction with their general health status. In another study, investigators reported that among 72 patients with knee OA, patients receiving 12 weeks of Tai Chi reported significantly less pain and stiffness than patients receiving routine treatment. In addition, physical functioning, balance and abdominal muscle strength were significantly improved within the Tai Chi group [15].

In a three-armed, randomized trial of 152 older patients with chronic symptomatic hip and knee OA, researchers found that, when compared with a waiting-list control group, both 12-week Tai Chi classes and hydrotherapy classes provided large and sustained improvements in physical function. All significant improvements were sustained at 24 weeks [16]. A six-week group Tai Chi program, followed by six weeks of home Tai Chi training, showed significant improvements in knee pain and physical function compared with an attention control in 41 elderly patients with knee OA. However, the benefits for knee pain scores were not sustained throughout the follow-up detraining period (weeks 13–18) [17]. A single-blind, randomized trial of 40 patients showed that patients randomized to 12 weeks of Tai Chi exhibited significantly greater improvements in pain, physical function, depression, self-efficacy and health status compared with the attention controls. Patients who continued Tai Chi practice after 12 weeks reported durable benefits in pain and function [18]. A recent randomized, controlled trial of 82 women with OA suggested that six months of Tai Chi exercise significantly improved knee extensor endurance and bone mineral density and decreased patients’ fear of falling, compared with a self-help education program [19]. Similar positive findings showing short-and long-term benefits of Tai Chi practice have been well-documented in both randomized controlled clinical trials and observational studies, including improvements in balance control, flexibility, muscular strength and endurance—benefits important for patients with symptomatic OA.

Positive results in balance improvement and functional capacity were also reported for an older population with Parkinson’s disease [20]. In a 24-week randomized trial comparing Tai Chi with a resistance-training program or a stretching program, Tai Chi was effective in improving postural stability and other functional outcomes in patients with mild to moderate Parkinson’s disease.

Improvements in primary and secondary outcomes were maintained 3 months after the intervention. Consistent with prior research, these findings suggest that Tai Chi training appears to substantially improve strength and functional capacity for older adults with gait dysfunction. Tai Chi may confer these benefits to individuals with OA-associated gait dysfunction as well.

To further systematically quantify the effects of Tai Chi for patients with OA, researchers conducted a recent systematic review and meta-analysis of randomized, controlled trials to determine the effectiveness of Tai Chi mind-body therapy on symptomatic knee OA [21]. Of the 458 potentially relevant studies published between 2000 and 2009 that were identified, 6 with a total of 382 subjects (80% of whom were women) met the inclusion criteria.The sample sizes varied between 33 and 152. The mean age was 68 years and the treatment durations ranged from 8 to 12 weeks. The duration of OA ranged from 6 to 11 years. A pooled effect size was −0.72 (95% CI −0.97, −0.47) favouring Tai Chi with a heterogeneity score (I^2^) of 0%. Overall, the results from this meta-analysis suggest that Tai Chi training may provide an ideal form of exercise for older individuals with symptomatic knee OA.

Taken as a whole, the evidence is promising and suggests that Tai Chi training may provide an ideal form of exercise for older individuals experiencing OA-related pain, dysfunction or disability. As a form of physical exercise, Tai Chi may enhance cardiovascular function, muscular strength, proprioceptive acuity and neuromuscular activity, and may promote the integration of mind and body to reduce pain. Stronger muscles and better balance coordination can also improve physical function and stability of joints. Increased periarticular muscle strength may protect joints from traumatic impacts. Additionally, improving self-efficacy, social function and depression can help people build confidence, seek support and overcome fear of pain leading to improved physical, psychological and psychosocial well-being and overall quality of life.

## 4. Practical Aspects of Accessing CAM Treatments

Evidence suggests that acceptance of CAM therapies, particularly Tai Chi and acupuncture, is growing. Patients and providers alike are increasingly interested in CAM therapies because of their potential as an effective remedy for reducing pain while improving physical and psychological health and well-being. The three principle barriers preventing patients from accessing these CAM treatments in the United States are uncertainties about the methods, costs and the logistics of service procurement. Thus, the evidence behind these treatments must be discussed fairly with patients so that they can make an informed decision.

Out-of-pocket costs for CAM services can be significant and often are not covered by insurance. Still, many plans are beginning to include some discounts or rebates for these services as the evidence for their efficacy grows. Providers should encourage patients to check with their insurance plans.

Finding an experienced CAM provider can be difficult. A growing number of physicians are receiving dual training in both western and eastern medicine, which is beneficial for patients uncertain of how to integrate the two philosophies. When selecting a provider offering CAM treatments, the most important characteristics for patients to consider are the provider’s general experience and their experience treating musculoskeletal disorders. Tai Chi and acupuncture should not be a replacement for conventional care or be used to postpone seeing a physician about a medical condition.

Standards for training Tai Chi instructors do not exist, so providing patients with access to experienced instructors is essential. Ideally, practitioners should have five to ten years of experience working with arthritis patients. Additionally, patients should inquire as to how the therapy might be modified to accommodate their conditions. For example, some forms of Tai Chi require deep knee bending which could be painful and potentially harmful to patients with knee OA. An experienced provider will be able to adapt their Tai Chi principles in a safe manner while preserving the effect and philosophy of the therapy.

As the demand and evidence for CAM therapies grow, educating health care providers and patients about the clinical implications of these remedies is vital. By providing practical information about methods, costs and experience, providers can effectively encourage their patients to explore the options of integrating western and eastern medicines.

## 5. Summary

In summary, the pathophysiological basis of OA is complex and multifaceted, and symptomatic OA is heterogeneous. Emerging evidence from clinical trials suggest that both acupuncture and Tai Chi mind-body therapies are safe and effective treatments for OA. Integrative approaches combine the best of conventional medicine and CAM therapies to ultimately improve patient care. These modalities may lead to the development of better disease-modifying strategies that could improve symptoms and decrease OA progression.

## References

[1] Felson DT (2006). Clinical Practice; Osteoarthritis of the Knee. N Engl J Med.

[2] Barnes PM, Bloom B, Nahin Rl (2007). Complementary and Alternative Medicine Use Among Adults and Children. Natl Health Stat Report.

[3] NIH Consensus Conference (1998). Acupuncture. JAMA.

[4] Sherman KJ, Cherkin DC, Eisenberg DM, Erro J, Hrbek A, Deyo RA (2005). The Practice of Acupuncture, Who Are the Providers and What Do They Do?. Ann Fam Med.

[5] Berman BM, Lao L, Langenberg P, Lee WL, Gilpin AM, Hochberg MC (2004). Effectiveness of Acupuncture as Adjunctive Therapy in Osteoarthritis of the Knee: A Randomized, Controlled Trial. Ann Intern Med.

[6] Witt CM, Jena S, Brinkhaus B, Liecker B, Wegscheider K, Willich SN (2006). Acupuncture in Patients with Osteoarthritis of the Knee or Hip: A Randomized, Controlled Trial with an Additional Nonrandomized Arm. Arthritis Rheum.

[7] Scharf HP, Mansmann U, Streitberger K, Witte S, Karmer J, Maier C (2006). Acupuncture and Knee Osteoarthritis: A Three-armed Randomized Trial. Ann Intern Med.

[8] Suarez-Almazor ME, Looney C, Liu Y, Cox V, Marcus DM (2010). A Randomized Controlled Trial of Acupuncture for Osteoarthritis of the Knee: Effects of Patient-provider Communication. Arthritis Care Res(Hoboken).

[9] White A, Foster NE, Cummings M, Barlas P (2007). Acupuncture Treatment for Chronic Knee Pain: A Systematic Review. Rheumatology (Oxford).

[10] Manheimer E, Cheng K, Linde K, Lao L, Yoo J (2010). Acupuncture for Peripheral Joint Osteoarthritis. Cochrane Database Syst Rev.

[11] Manheimer E, Linde K, Lao L, Bouter LM, Berman BM (2007). Meta-analysis: Acupuncture for Osteoarthritis of the Knee. Ann Intern Med.

[12] Vickers AJ, Cronin AM, Maschino AC, Lewith G, Macpherson H, Foster NA (2012). Acupuncture for Chronic Pain. Arch Inter Med.

[13] Wang C, Collet JP, Lau J (2004). The Effect of Tai Chi on Health Outcomes in Patients with Chronic Conditions: A Systematic Review. Arch Inter Med.

[14] Hartman CA, Manos TM, Winter C, Hartman DM, Li B, Smith JC (2000). Effects of T’ai Chi Training on Function and Quality of Life Indicators in Older Adults with Osteoarthritis. J Am Geriatr Soc.

[15] Song R, Lee EO, Lam P, Bae SC (2003). Effects of Tai Chi Exercise on Pain, Balance, Muscle Strength, andPerceived Difficulties in Physical Functioning in Older Women with Osteoarthritis: A Randomized Clinical Trial. J Rheumatol.

[16] Fransen M, Nairn L, Winstanley J, Lam P, Edmonds J (2007). Physical Activity for Osteoarthritis Management: A Randomized Controlled Clinical Trial Evaluating Hydrotherapy or Tai Chi Classes. Arthritis Rheum.

[17] Brismee JM, Paige RL, Chyu MC, Boatright JD, Hagar JM (2007). Group and Home-based Tai Chi inElderly Subjects with Knee Osteoarthritis: A Randomized Controlled Trial. Clin Rehabil.

[18] Wang C, Schmid CH, Hibberd PL, Kalish R, Roubenoff R (2009). Tai Chi is Effective in Treating Knee Osteoarthritis: A Randomized Controlled Trial. Arthritis Rheum.

[19] Song R, Roberts BL, Lee EO, Lam P, Bae SC (2012). A Randomized Study of the Effects of T’ai Chi on Muscle Strength, Bone Mineral Density, and Fear of Falling in Women with Osteoarthritis. J Altern Complement Med.

[20] Li FZ, Harmer P, Fitzgerald K (2012). Tai Chi and Postural Stability in Patients with Parkinson’s Disease. N Engl J Med.

[21] Bannuru RR, Samuel A, Wang C (2012). How Effective is Tai Chi Mind-body Therapy for Knee Osteoarthritis? A Systematic Review and Meta-Analysis. Osteoarthritis and Cartilage.

